# Custom-made 3D-printed boot as a model of disuse-induced atrophy in murine skeletal muscle

**DOI:** 10.1371/journal.pone.0304380

**Published:** 2024-05-31

**Authors:** Giulio Masiero, Giulia Ferrarese, Eleonora Perazzolo, Martina Baraldo, Leonardo Nogara, Caterina Tezze

**Affiliations:** 1 Department of Biomedical Science, University of Padova, Padova, Italy; 2 Veneto Institute of Molecular Medicine, Padova, Italy; 3 Biozentrum, University of Basel, Basel, Switzerland; 4 Department of Pharmaceutical and Pharmacological Sciences, University of Padua, Padua, Italy; University of Minnesota Medical School, UNITED STATES

## Abstract

Skeletal muscle atrophy is characterized by a decrease in muscle mass and strength caused by an imbalance in protein synthesis and degradation. This process naturally occurs upon reduced or absent physical activity, often related to illness, forced bed rest, or unhealthy lifestyles. Currently, no treatment is available for atrophy, and it can only be prevented by overloading exercise, causing severe problems for patients who cannot exercise due to chronic diseases, disabilities, or being bedridden. The two murine models commonly used to induce muscle atrophy are hindlimb suspension and ankle joint immobilization, both of which come with criticalities. The lack of treatments and the relevance of this atrophic process require a unilateral, safe, and robust model to induce muscle atrophy. In this work, we designed and developed a 3D-printed cast to be used for the study of disuse skeletal muscle atrophy. Applying two halves of the cast is non-invasive, producing little to no swelling or skin damage. The application of the cast induces, in 2-weeks immobilized leg, the activation of atrophy-related genes, causing a muscle weight loss up to 25% in the gastrocnemius muscle, and 31% in the soleus muscle of the immobilized leg compared to the control leg. The cross-sectional area of the fibers is decreased by 31% and 34% respectively, with a peculiar effect on fiber types. In the immobilized gastrocnemius, absolute muscle force is reduced by 38%, while normalized force is reduced by 16%. The contralateral leg did not show signs of overload or hypertrophy when compared to free roaming littermates, offering a good internal control over the immobilized limb. Upon removing the cast, the mice effectively recovered mass and force in 3 weeks.

## Introduction

In humans, skeletal muscle atrophy is a condition characterized by a decrease in muscle mass and strength caused by an imbalance in protein synthesis and protein degradation [1–4]. This condition can be caused by reduced or absent physical activity or aging, and it is often related to illness, forced bed rest, or unhealthy lifestyles; it is closely linked to sarcopenia, the age-related decline in muscle mass and strength [5, 6]. The presence of low muscle mass and function during acute illness is associated with increased health economic costs in terms of increased length of hospital stay, rehabilitation costs and the need for institutional care or social care on discharge [7]. Preventing atrophy will have wider economic and individual benefits to patients. Treatment strategies for atrophy include non-pharmacological interventions such as lifestyle management, physical exercise, and adequate calorie and protein intake as first-line therapies [8]. These recommendations are not easily accessible to all patients for various reasons; therefore, their therapeutic impact is limited or poorly effective. In this context, it is important to develop animal models that allow the study of the atrophic process to uncover therapeutic vulnerabilities and possible innovative treatments [9–12]. Various studies on muscle wasting are based on animal models in which muscle homeostasis is altered by systemic or multi-organ conditions, such as cachexia, fasting, or denervation [13, 14]. Studying disease-induced muscle atrophy using these models is appropriate in a systemic context. Still, the combination of several factors may prevent the identification of muscle-specific mechanisms that cause atrophy. Murine models of disuse-induced muscle atrophy are important as a translational approach for developing new therapeutical interventions for treating muscle atrophy. The disuse models commonly adopted include hindlimb suspension and ankle joint immobilization [1, 15]. These techniques have been widely used in many animal models, mostly involving rats and mice. Additionally, other animals models have been subjected to disuse experiments, like rabbits [16, 17], chickens [18], and dogs [19, 20].

The hindlimb suspension is achieved by mounting the tail of the animal on an elevated rail so that the posterior limbs do not touch the ground and its body weight is supported by the structure, thus preventing the muscles from exerting their physiological functions [21]. Ankle joint immobilization, on the other hand, restricts the free movement of a single posterior limb by blocking the dorsiflexion of plantar flexion of the foot. The immobilized joint does not allow the change in length of the front and back of the lower limb; thus, the animal can no longer properly load the leg with its body weight, often causing dragging of the immobilized limb or simply adopting three-legged gait [22, 23]. Hindlimb suspension methods reported variable degrees of muscle loss: in 3 days, 13% of atrophy has been reported [24], 21% after 7 days [25], 10% after 14 days [26]. Ankle joint immobilization is better characterized in the rat model [27], while in mice, studies of immobilization without recovery for 7 days [28], 14 days [27, 29] and 28 days [27] have been performed, reporting a degree of muscle loss ranging from 10% [28] to 22% [29]. This muscle loss is mainly associated with inflammation and collagen deposition.

In addition to the described models, animals exposed to microgravity or space flight have also been studied recently. In Sandonà et al. [30] mice have been in space for 91 days exhibited remarkable atrophy of the soleus muscle, while left EDL muscle unaffected [31–33].

Hindlimb suspension has the advantage of preserving the integrity of the animal, but it also induces atrophy in both limbs, depriving of the possibility of using the contralateral muscles as an internal control, so it resembles more closely microgravity conditions [34].

Other negative aspects are the exposure to a hemodynamic stress and the distress caused by single-caged housing of social animals [35].

Joint immobilization techniques are usually invasive or require methods that can damage the skin, thus inducing inflammatory responses that would necessarily alter the atrophic processes; these complications would also limit the possibility of performing follow-up recovery studies. Not least, the muscle functionality is not usually analyzed in the already published methods. Here, we propose a new model for inducing skeletal muscle atrophy using a 3D-printed cast. The cast can be applied to a single limb in a non-invasive way and produces little to no swelling and skin damage while inducing a very consistent muscle atrophy (measured in terms of wet muscle weight and cross-sectional area) in two weeks and a suppressed muscle force. The device easily allows the study of the reloading phase by removing it from the animal. With this new method, we intend to provide a reliable, non-invasive, and highly reproducible way to study disuse skeletal muscle atrophy in animal models.

## Materials and methods

### Study approval

Animals were handled by specialized personnel under the control of inspectors of the Veterinary Service of the Local Sanitary Service (ASL 16—Padova) and the local officers of the Ministry of Health. Animal experiments were conducted according to the Guide for the Care and Use of Laboratory Animals (NIH; National Academies Press, 2011), as well as the Italian law for the welfare of animals. The Italian Ministero della Salute approved all animal experiments, Ufficio VI (Rome, Italy; authorization number 448/2021 PR).

### Experimental model

All experiments were performed on 3-month-old male C57BL/6 mice. Mice were housed in independent cages in an environmentally controlled room (23°C, 12-h-light-dark cycle) with ad libitum access to food and water. Gastrocnemius, tibialis anterior, and soleus muscles were collected at different time points: after 1 (10 mice) and 2 weeks (8 mice) of unilateral immobilization and after 1 (3 mice) and 3 (8 mice) weeks of recovery after disuse. Muscles of control mice (8 mice not immobilized) were collected from age matched mice. The muscles were removed at the described time points and snap-frozen in liquid nitrogen for subsequent analyses.

### 3D printing details

The boot model was designed using Autodesk Fusion 360® (academic license own by LN). The model was sliced using Ultimaker Cura 3D software (version 5.2) and the print was conducted using a common Creality Ender 3 Pro, using polylactic acid polymer (PLA). The first layers are of different colors to identify the size, while the body is printed in transparent-opaque plastic to be able to see the skin underneath. The model has been printed using a 0.4mm nozzle regulated at a temperature of 195°C, bed temperature of 60°, at a speed of 40mm/s. The layer height (z-axis resolution) has been set to 0.1mm, and the cooling fan is set at 100% from layer 2. The two halves of the boot are printed with the flat edges facing the bed, without support and without infill. The total weight of the two halves combined ranges from 1.036±0.003g to 1.489±0.009g (mean±SD) depending on the printing size, respectively 85 to 100% of the designed model. After the printing, each half of the device was smoothed by hand using thin sandpaper and inspected under a stereomicroscope to ensure no leftover sharp plastic bits could harm or discomfort the animal. The STL file and printing details will be made available by the authors.

## Immobilization method

**Fig 1:**
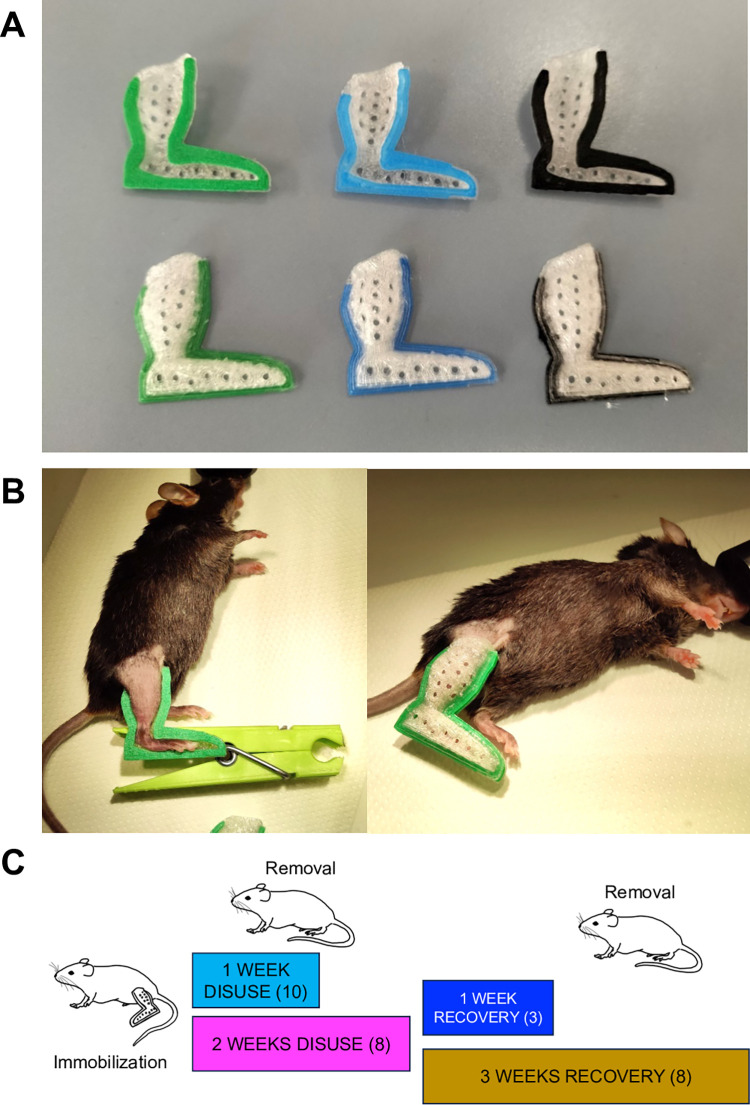
3D printed-cast immobilization procedure. **(A)** Picture of the two halves of different boots seizes. **(B)** Hindlimb immobilization procedure on a mouse anesthetized with isoflurane. **(C)** Time schedule of experimental groups with corresponding color code. Mouse draw adapted from BioRender.com (2020) licensed to University of Padova.

Mice were anesthetized by inhalation of 3% isoflurane enriched with oxygen, and the right leg was shaved. The two halves of the boot were adjusted on the hindlimb and glued together using a few drops of super glue. The glue was poured through the gap between the two plastic surfaces at the edge of the boot without letting it reach the skin underneath. A modified clothespin (Fig 1B) was used to keep the parts in the correct position during the glue curing time (see S1 Video). The complete procedure lasts no longer than 10 minutes. The device was designed and positioned to maintain knee joint extension while restricting ankle torsion. The blocked leg is referred to as the “immobilized leg” in the main text, while the other hindlimb has been used as a contralateral comparison and is referred to as the “free leg” throughout this manuscript.

Animals were immobilized for 1 or 2 weeks, 2 groups were sacrificed respectively at the end of each period, and muscles were harvested and weighed (Fig 1C). The two remaining groups were used to study muscle recovery and were sacrificed 1 and 3 weeks after the end of the 2 weeks; muscles were collected and weighed. A group of free-roaming (control) animals has also been analyzed at the same time points to be compared with the free leg data. The unmounting process and behavior of free roaming restricted animals have been recorded and are available in the supplementary material (see S2 and S3 Videos). Precision side-cutting nippers were used to sunder the two halves of the boot, by clipping the tip where the foot does not reach. The plastic was rigid enough that the two halves come apart quite easily, without exerting any lever or mechanical stress on the leg.

### In vivo force measurements

Immediately after removing the boot, the *in vivo* muscle force was measured as previously described [36, 37] in both legs (immobilized and control). Briefly, 8 animals per condition were deeply anesthetized, and the foot was mounted on a 305B muscle lever system (Aurora Scientific, ON, Canada). The common peroneal nerve was cut to prevent recruitment of the tibialis anterior muscle. The knee was stabilized, and an electrical stimulation was applied to the sciatic nerve, inducing the isometric plantar flexion of the foot due to gastrocnemius muscle activation. A force-frequency curve was obtained by stimulating at increasing frequencies (starting with a single depolarization up to 150Hz, single train duration 200ms, pulse width 200μs, 6-8V). Animals were then sacrificed by cervical dislocation according to the approved animal protocols, and muscles were dissected, weighed, and frozen. Force was normalized to the weight of the gastrocnemius to estimate specific force. Experimental data were analyzed using a custom program compiled in LabView [38].

### Gait analysis

A total of 8 adult male mice (3 months old) were recorded walking on a treadmill at 10cm/s from a ventral point of view (see S4 Video). The animals were tested following 2 weeks of immobilization; the plastic boots were removed, and mice were allowed to adapt to the treadmill before the test. The machine and the software used to analyze the gait were the DigiGait setup [39]. The parameters extracted were: swing duration, % swing stride, brake duration, % brake stride, propel duration, % propel stride, stance duration, % stance stride, stride duration, % brake stance, % propel stance, stride length, stride frequency, stance/swing ratio, absolute paw angle, number of steps, paw area at peak stance, % of shared stance, overlap distance, paw placement position, midline distance, and paw drag.

### Histology

Thin 10 μm cryosections of the gastrocnemius, tibialis anterior, and soleus muscles from different time points (2 weeks of disuse, 1 and 3 weeks of recovery) were stained using Hematoxylin (Harris Hematoxylin solution, HHS16, Sigma Aldrich) and Eosin (Eosin Y solution alcoholic, HT110116, Sigma Aldrich). The slides were immersed in Hematoxylin solution for 6 minutes and then washed with tap water for 3 minutes. Subsequently, the slides were immersed in alcoholic acid (99% of ethanol 70% and 1% hydrogen chloride 258148, Sigma Aldrich) for 10 seconds and washed with tap water for 3 minutes. The slides were immersed in Eosin for 2 minutes, and the tissues were dehydrated in alcohols in increasing concentration, 70%, 95% and 100%, respectively, for 5, 2 and 3 minutes (Ethanol puriss.p.a., absolute, ≥99.8% (GC), 32221, Sigma Aldrich). Finally, the slides were immersed in Xylene (534056, Sigma Aldrich) for 10 minutes, and the coverslip was mounted with a Balsamo Eukitt mounting medium (09–00502, Bio-optica). Sections were imaged using a Leica DM6B direct fluorescent microscope equipped with an RGB digital camera (Leica DFC 7000 T) in a bright field with 40x of magnification (HCX PL FLUOTAR 40x/0.75 dry) at room temperature. The acquisition software used was LAS X 3.7.0.20979.

### Immunostaining

Thin 10 μm cryosections of the gastrocnemius, tibialis anterior, and soleus muscles from different time points (1 and 2 weeks of disuse, 1 and 3 weeks of recovery) were cut at the middle of the muscle length. Anti-Dystrophin I antibody (rabbit, Abcam ab15277, dilution 1:100) was incubated overnight at 4°C to identify the sarcolemma. The next day, the secondary antibodies (anti-rabbit Cy3, Jackson 111-165-003, dilution 1:200) was incubated for 1 hour at room temperature to measure the cross-sectional area (CSA). The antibody Anti-Neural Cell Adhesion Molecule (rabbit, Merck AB5032, dilution 1:100) was used to identify denervated fibers. Sections were fixed for 10 minutes in ice-cold methanol (puriss. p.a,≥9.8%, 32213, Sigma Aldrich) and NCAM I antibody (rabbit, Merck AB5032, dilution 1:100) was incubated overnight at 4°C. The next day, the secondary antibodies (anti-rabbit Cy3, Jackson 111-165-003, dilution 1:200) was incubated for 1h at room temperature. For the myosin isoform identification, slices were blocked with MOM (Vector Biolab BMK-2202) for 40 minutes at 37°C, incubated with primary antibodies (BA-D5 for type 1 fibers, SC-71 for type IIA fibers, BF-F3 for type IIB fibers, no stain for IIX fibers, developed by Prof. Stefano Schiaffino was obtained from the Developmental Studies Hybridoma Bank, created by the NICHD of the NIH and maintained at The University of Iowa, Department of Biology, Iowa City, IA 52242) [40] at 4°C overnight. The corresponding secondary antibodies (Jackson Dy405 115-475-207, AF488 115-545-205, AF549 115-587-020 fragment affinity purified) were incubated at 1:100, 1:200 and 1:200 for 1h at 37°C. The cryosection images of the gastrocnemius muscles were acquired at 10× magnification (objective lens: HC PL FLUOTAR 10x/0.30 dry) or 20x magnification (objective lens: HC PL FLUOTAR 20x/0.50 dry) using a motorized table Leica DM6B direct fluorescent microscope equipped with a digital camera (Leica DFC 7000 T, acquisition software LAS X 3.7.0.20979) in the appropriate emission channels at room temperature. All analysis was performed on complete muscle slices, stitched by the Leica software. The morphometric analyses (CSA) were made using MATLAB Semi-Automatic Muscle Analysis using Segmentation of Histology (SMASH) software [41], while myosin isoform analysis was performed using FIJI [42].

### Real-time quantitative PCR

Total RNA was extracted from gastrocnemius muscles at 1 week of immobilization using TRIzol (15596018, Invitrogen). Complementary DNA was generated from 400ng of RNA reverse transcribed with SuperScript III Reverse Transcriptase (18080093, Invitrogen). Duplicates of cDNA samples were then amplified on the 7900HT Fast Real-Time PCR System (Applied Biosystems) using the Power SYBR Green RT-PCR kit (4367659, Applied Biosystems). All data were normalized to GAPDH expression and plotted in arbitrary units as mean ± SEM.

The oligonucleotide primer sequences are reported below:

mMURF1 Fw ACCTGCTGGTGGAAAACATC

mMURF1 Rv ACCTGCTGGTGGAAAACATC

mMUSA1 (MfbxO30) Fw TCGTGGAATGGTAATCTTGC

mMUSA1 (MfbxO30) Rv CCTCCCGTTTCTCTATCACGmAtrogin1 Fw GCAAACACTGCCACATTCTCTC

mAtrogin1 Rv CTTGAGGGGAAAGTGAGACG

mActin Fw CTGGCTCCTAGCACCATGA

mActin Rv GGTGGACAGTGAGGCCAGGmTBP Fw TCATTTTCTCCGCAGTGCCC

mTBP Rv CCAAGCCCTGAGCATAAGGT

### Statistics

Statistical analysis was performed using GraphPad Prism 8 (GraphPad Software, San Diego, California USA, www.graphpad.com). The data have been analyzed using two-tailed Student t-test comparisons between the free hindlimb and the free-roaming wild type (wt) to assess nonsignificant alterations of the measured outcome. Thus, comparison of the immobilized hindlimb with the free contralateral one is performed using paired 2-tailed Student t-test. Dot plots are presented as mean±SEM. Bar plots are presented as mean±SD. *P* values below 0.05 were considered significant.

### Online supplemental material

S1 Fig contains muscle weight of the tibialis anterior of control, free leg, and blocked leg at 2 weeks of immobilization, and immunofluorescence pictures of muscle sections stained for NCAM. S2 Fig contains all the results of the DigiGait® analysis that were not included in Fig 6. S1 Video contains the boot mounting procedure, S2 Video contains the boot unmounting procedure, S3 Video contains free-roaming immobilized animals and S4 Video is composed by individual frames taken with by the DigiGait® setup to assess the walking capability of an animal after boot removal at 2 weeks of immobilization.

## Results

### 3D printed cast immobilization procedure

We present here a new model to induce and study skeletal muscle atrophy in mice. We designed a 3D-printable plastic boot (Fig 1A) that can be applied to the hindlimb of a mouse without causing any tissue alteration but effectively prevents the loading of the leg, therefore inducing atrophy. Mice were anesthetized, the chosen leg was shaved, and the two halves of the boot were glued together (Fig 1B and see S1 Video). Muscle atrophy under conditions of immobilization varies significantly depending on the degree to which the joint is restricted from movement (e.g., casting versus pinning) and the angle at which the joint is fixed; muscle atrophy is greatest when a muscle is immobilized in a shortened/neutral position versus a lengthened position [43]. We secured the ankle to keep the foot at a 90° angle with the leg to achieve a neutral position for all the muscles. (Fig 1B). The 3D printed boot is made of hard plastic, custom-designed, and carefully tailored to ensure a snug yet unrestrictive fit around the ankle region. The boot is adjusted to limit ankle movement, blocking both dorsiflexion and plantarflexion. This double restriction effectively immobilizes the ankle joint, thus inducing the desired condition for studying muscle atrophy.

### Comprehensive muscle atrophy following 2 weeks of unilateral immobilization

**Fig 2:**
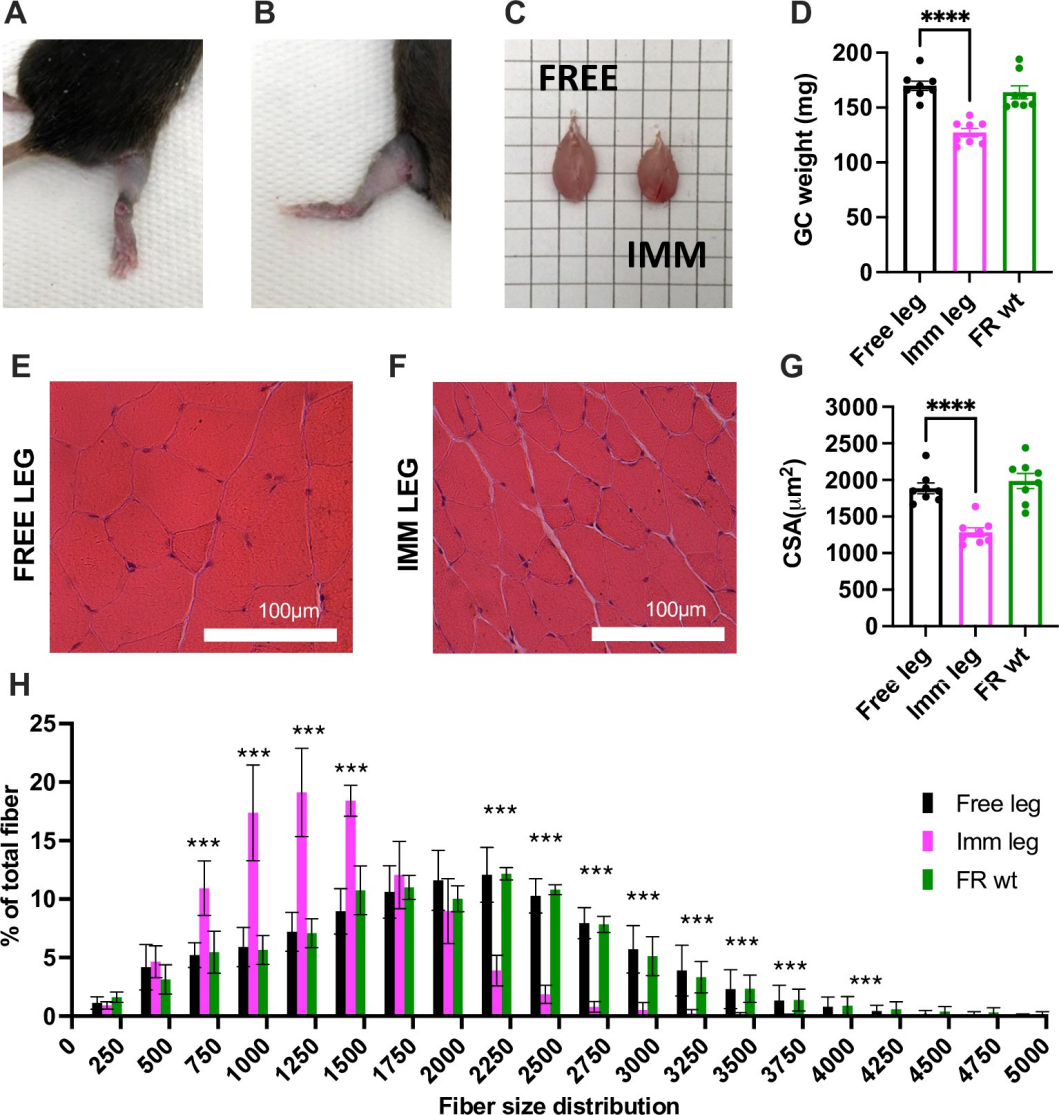
Comprehensive muscle atrophy following two weeks of unilateral immobilization. **(A-B)** After two weeks of disuse, the immobilized leg did not display edema, and the skin appeared intact. **(C)** Comparison between the gastrocnemius muscle of the free leg (left) and the contralateral muscle of the immobilized leg (right). **(D)** Comparison between free, immobilized, control gastrocnemius wet weight. **(E)** Representative images of Hematoxylin and Eosin of free and **(F)** immobilized gastrocnemius muscles. Scale bar = 100μm. **(G)** Myofibers cross-sectional area analysis of free, immobilized, control gastrocnemius. **(H)** the relative fiber size distribution. (leg n = 8 per each condition, mean±SD). Statistical significance was calculated using paired two-tailed Student’s t test. Data are mean±SEM (A to G), mean±SD (H). ** p<0,01; *** p<0,001; **** p<0,0001.

The designed boots were applied to 3-month-old wild-type mice and removed after 2 weeks of immobilization (see S1 and S2 Videos, see also timeline of experimental method Fig 1C). The 2-week time point has been decided based on the available literature covering different immobilization protocols or devices [29, 44, 45] to compare our new method with those already available. Upon boot removal, the skin of the animals appeared overall intact (Fig 2A and 2B and see S3 Video), and the gastrocnemius (GC) muscles belonging to the immobilized legs were already visibly atrophic, but devoid of collateral tissue alterations (Fig 2C).

Following a 2-week immobilization period, the gastrocnemius and tibialis anterior muscles exhibited a notable 25% reduction in weight in the immobilized leg compared to the free contralateral leg (Fig 2D and S1 Fig). This reduction is comparable to the control muscles of free-roaming animals (Fig 2D and S1 Fig). Additionally, it is noteworthy that the soleus muscle experienced a significant loss, amounting to 31% of its initial mass (S1 Fig). Histological analysis of muscle slices revealed that the immobilization period does not induce alteration in muscle architecture, inflammation, or degeneration to both free and immobilized leg muscles (Fig 2E and 2F).

Confirmed through cross-sectional area measurements, the atrophy is evident, with a notable 31% reduction in size for the gastrocnemius and a 34% reduction for the soleus. (Fig 2G and 2H; S1 Fig). In comparing the cross-sectional area and distribution of the free limb gastrocnemius with that of age- and sex-matched wild-type mice, it is noteworthy that no discernible differences were found. This underscores the absence of hypertrophy or compensation in the control leg compared to the leg of a free-roaming animal (Fig 2G and 2H).

### Comprehensive muscle weakening after 2 weeks of unilateral immobilization

**Fig 3:**
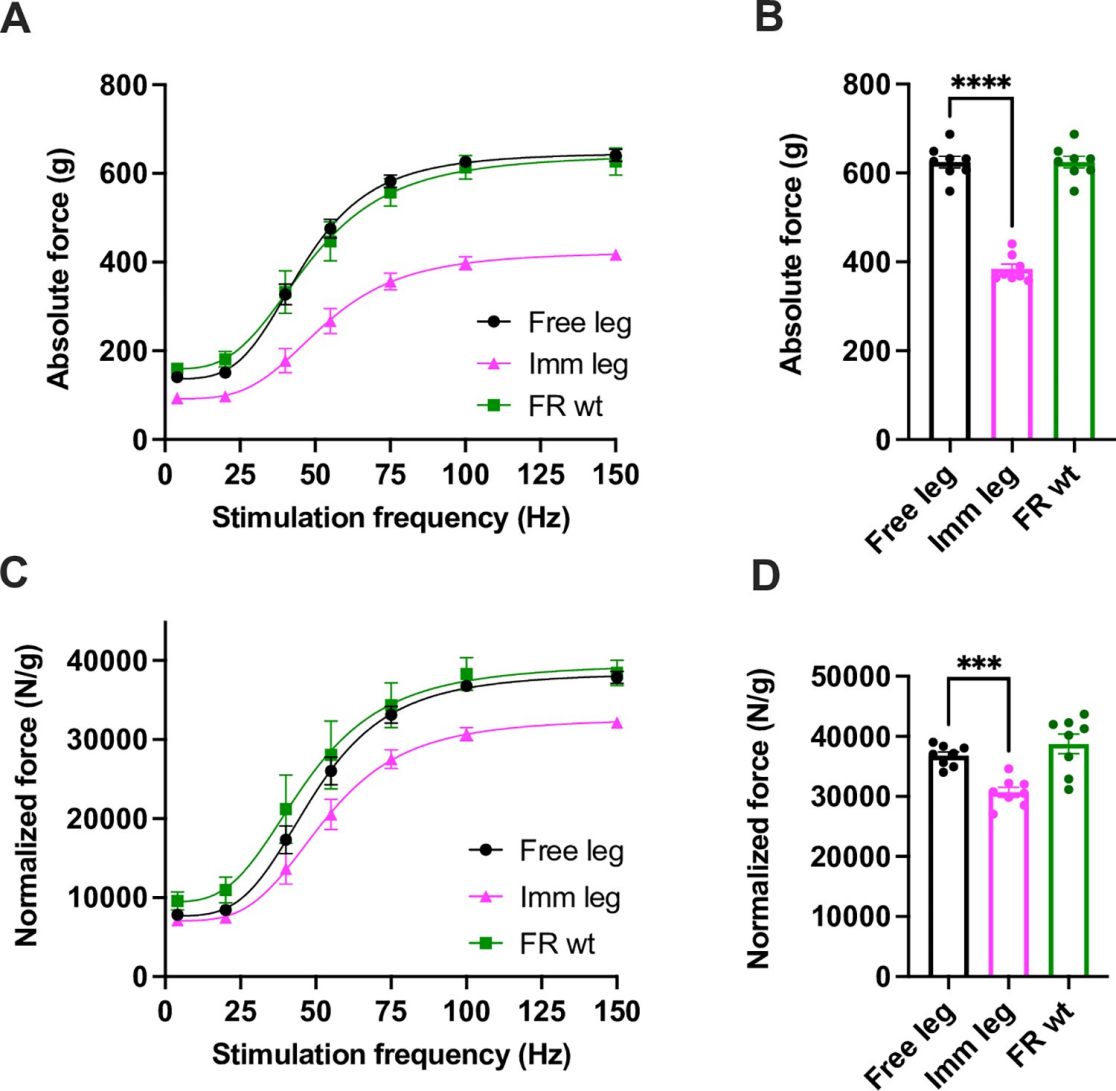
Comprehensive muscle weakening after 2 weeks of unilateral immobilization. Force-frequency curves were performed *in vivo* on gastrocnemius muscles. **(A-B)** Absolute force and **(C-D)** maximal specific force generated during tetanic contraction of control, free leg and immobilized one. (leg n = 8 per each condition) Statistical significance was calculated using paired two-tailed Student’s *t* test. Data are mean ± SEM, ** p < 0,01; *** p< 0,001, ****p<0,0001.

In previously published methodologies, the emphasis has primarily been on mass loss, with limited correlation to strength decline. In contrast, our primary objective is to evaluate atrophy and functional loss during disuse and reloading phases. Our particular interest lies in offering a precise and reproducible method to measure gastrocnemius muscle force in vivo, making this muscle our primary focus. Concerning functionality, the gastrocnemius muscle weight loss was accompanied by a 38.5±2.7% reduction in maximal absolute force when stimulated at the highest frequency of 100Hz compared to control and free legs (Fig 3A and 3B). The specific force (the force normalized for muscle mass), was also significantly decreased by 16.5±2.7% (Fig 3C and 3D), suggesting that force generation is also impaired in addition to a decrease in myofilaments content. Importantly, no differences were found between the free leg and the legs of controls, both in terms of force kinetics and at a frequency of 100 Hz.

### Effects of a two-week immobilization period myosin fiber types, NMJ stability, and ubiquitin ligase involved

**Fig 4:**
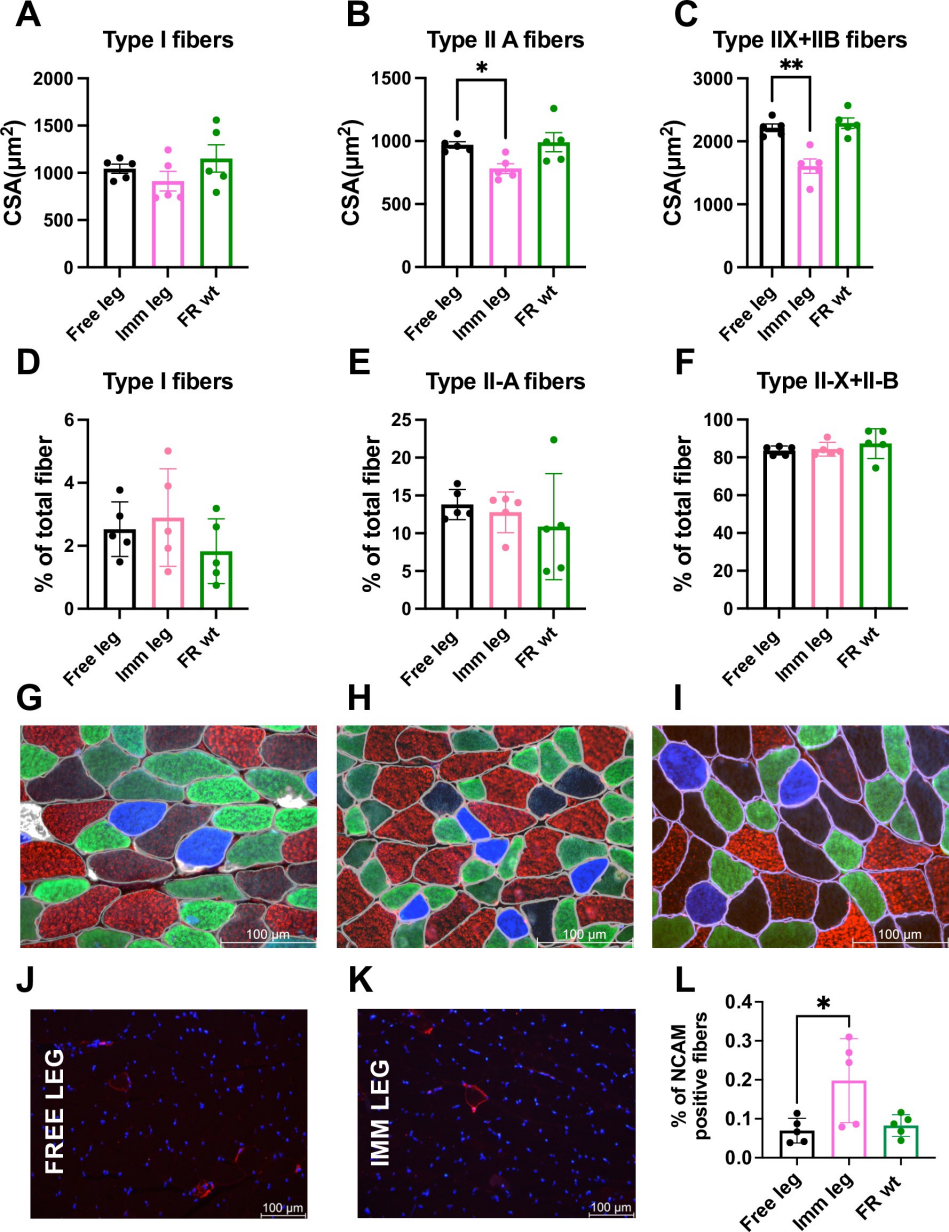
Effects of a two-week immobilization period on myosin fiber types of gastrocnemius muscle. **(A-C)** Comparison between free, immobilized, and free-roaming wild type legs of cross-sectional area variations according to fiber type and **(D-F)** of fiber type composition of the muscle. **(G)** Detail of fiber type composition of a free leg gastrocnemius, **(H)** an immobilized leg gastrocnemius and **(I)** a free roaming wildtype gastrocnemius (type I blue, type IIA green, type IIX black, type IIB red, dystrophin grey). Scale bar = 100μm; leg n = 5; magnification = 40x. **(J)** Immunofluorescence representative picture of free leg gastrocnemius cryosection stained for NCAM (red) and DAPI (blue), magnification = 20x (n = 5). **(K)** Immunofluorescence representative picture of immobilized leg gastrocnemius cryosection stained for NCAM (red) and DAPI (blue), n = 5; magnification = 20x. **(L)** Quantification of NCAM positive fibers in gastrocnemius of free, immobilized and free roaming wt animals (n = 5). Statistical significance was calculated using two-tailed Student’s t test. Data are mean±SEM, * p<0,05 ** p<0,01.

The disuse induced by the described 3D printed boot specifically causes atrophy in IIA, IIX and IIB fibers of the gastrocnemius, while type I fibers remain unaffected (Fig 4A–4C). Additionally, there is an absence of observable shifts in myosin types during the disuse period (Fig 4D–4I). The soleus exhibits atrophy in both type I and IIA fibers (S1 Fig) and a notable transition of myosin isoforms from type I to type IIA (S1 Fig) in line with the already reported literature.

**Fig 5:**
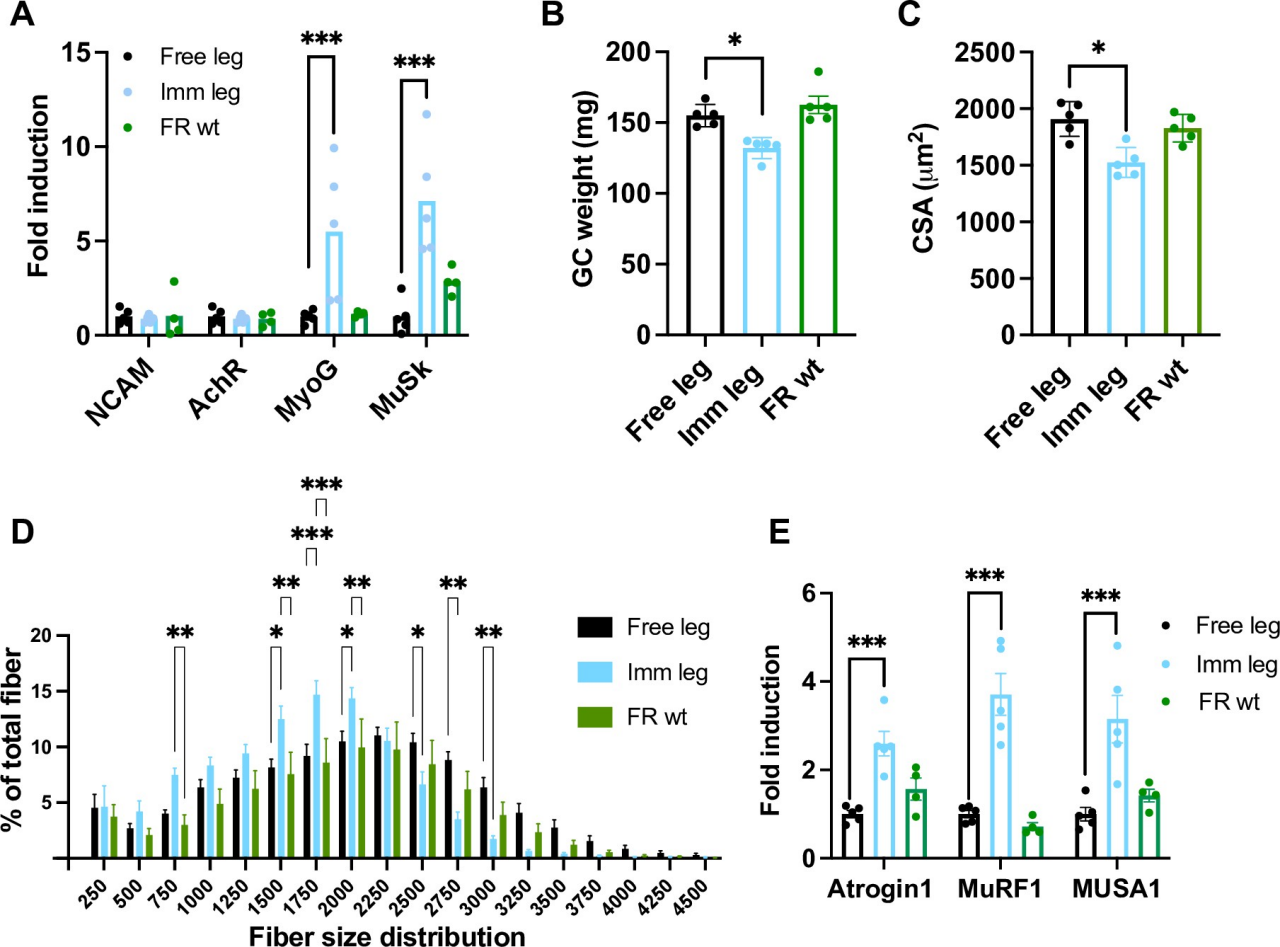
Effects of 1 week immobilization. **(A)** Quantitative RT-PCR of NJM genes expression in immobilized, free, and free roaming wild type leg after one week of immobilization, TBP normalized. **(B)** Comparison between free, immobilized, and free-roaming wild type legs of gastrocnemius muscle wet weight, **(C)** cross-sectional area and **(D)** relative fiber size distribution, and **(E)** Quantitative RT-PCR of atrogenes expression, TBP normalized (n = 5). Statistical significance was calculated using two-tailed Student’s t test. Data are mean±SEM (A, B, C, E), mean±SD (D). * p<0,05 ** p<0,01 *** p<0,001.

Another point to consider in the context of disuse is the instability of the neuromuscular junction. NCAM-positive fibers analysis showed a difference between immobilized and contralateral muscles or free roaming animal muscles after two weeks of immobilization in gastrocnemius (Fig 4J–4L). The same phenomenon is not observed in the soleus or tibialis anterior (S2 Fig). Concurrently, in the gastrocnemius after 1 week of disuse we conducted real-time PCR for genes associated with the neuromuscular junction instability, revealing induction specifically in MyoG and MuSk expression after 1 week of disuse (Fig 5A and S3 Fig). MuSk and MyoG, as documented in the literature, are primarily linked to the maintenance and growth of the neuromuscular junction, rather than denervation per se [46–48].

Finally, we have analyzed the expression of E3 ubiquitin ligase genes: Atrogin1, MuRF1, and MUSA1 (also known as Fbxo30). After the first week of disuse, gastrocnemius already lost 14% of mass (Fig 5B), corresponding to 20% of decrease in CSA (Fig 5C and 5D). The genes mentioned above were activated in the immobilized legs, indicating the presence of genetic pathways related to muscle atrophy (Fig 5E and S3 Fig). After two weeks of immobilization, the gene expression difference was no longer significant when comparing immobilized and free legs, suggesting that the atrophic program peaked within this time point (S3 Fig). We also confirmed that the atrogenes were not differentially expressed between the control animals and the free leg of immobilized mice (Fig 5E and S3 Fig).

### Reload capability

Recovering from prolonged muscle inactivity can be problematic in frail or old patients [49]. Therefore, the characterization of the recovery period following disuse skeletal muscle atrophy in animal models is very important.

For this reason, we investigated the recovery capability of immobilized mice with our boot model. A novel mouse-based model system developed for generating joint contracture using 3D-printed clamshell casts revealed measurable alterations in mouse gait [50]. The authors did not analyze muscle morphology, size, or function; rather, they aimed to create reversible and irreversible joint contractures in the knee and ankle.

Interestingly, the contractures affected the mice gait, which was analyzed using the DigiGait® analysis system [50]. Thus, to understand if our model might impede reloading in a similar manner, we recorded videos of mice walking on a treadmill after removing the plastic boots (see S4 Video) and extracted various gait-related parameters using the DigiGait setup and software [39].

**Fig 6:**
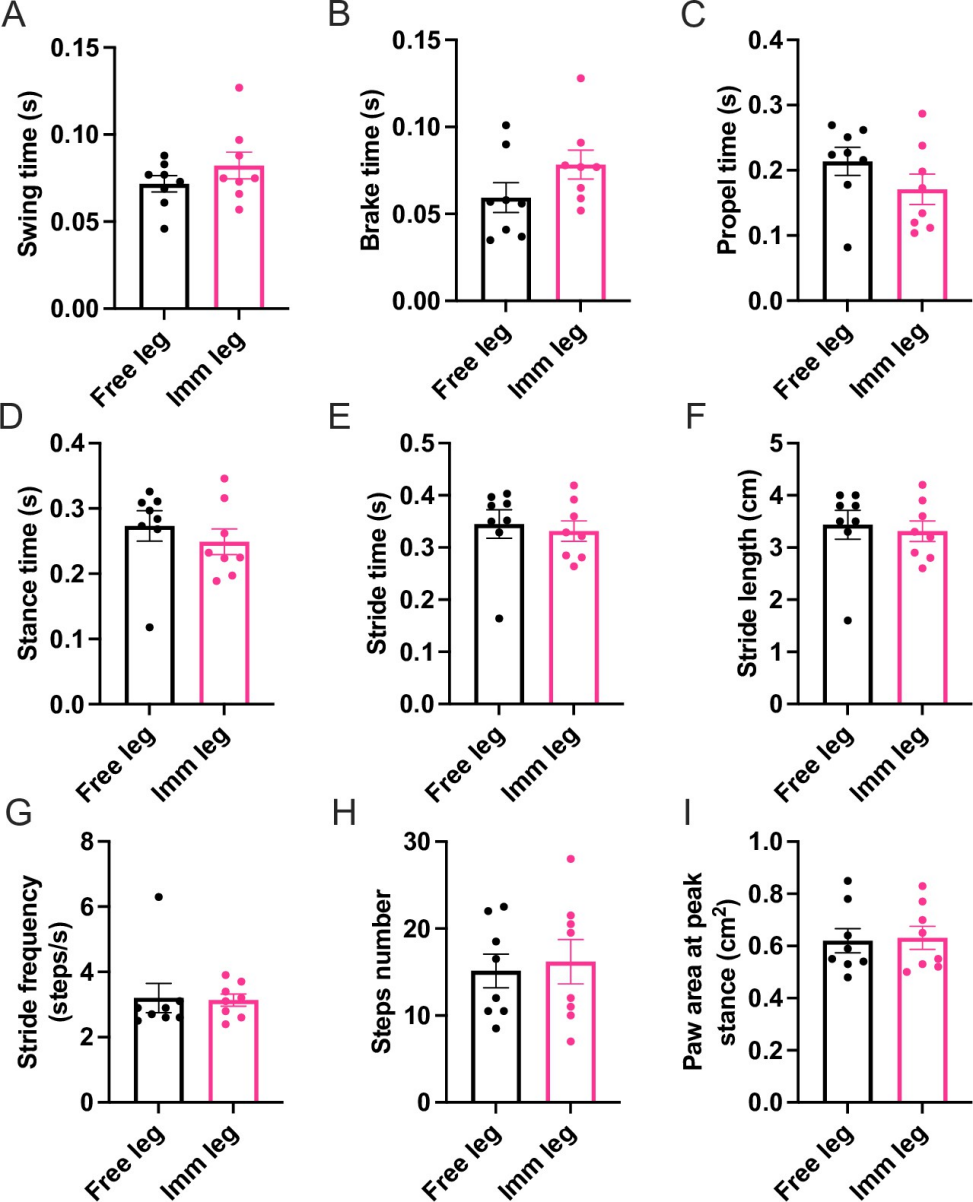
Digigait analysis. Gait is not impaired after 2 weeks of immobilization. Components of the stride: **(A)** swing, **(B)** brake, **(C)** propel, **(D)** stance and **(E)** overall stride. **(F)** Difference in length covered by stride. **(G)** Stride frequency. **(H)** Number of steps. **(I)** Area covered by the paw at peak stance (leg n = 8 per each condition). Statistical significance was calculated using paired two-tailed Student’s T-test. Data are mean±SEM.

The gait analysis showed no difference in all stride components (stance, swing, brake, propulsion) as well as the number of steps, the area covered by the paw at peak stance and stride duration, length, and frequency of hindlimbs (Fig 6 and S4 Fig), indicating symmetrical use of each hindlimb. There was no significant difference in the total stride length for each paw. This is also evident in the analysis of the individual stride components, which show that there is no difference in the time that each paw is in contact with the ground (stance), is lifted (swing), it approaches the ground (brake), and it is used to thrust forward (propel). The lack of difference in the number of steps taken by each leg and the frequency of the steps shows that the animals have no preference for using either leg. Finally, the lack of difference in the area covered by each foot at full stance proves that the animals are comfortable with loading normally both the free foot and the foot previously subjected to immobilization. Overall, we conclude that the mice could immediately reload the immobilized leg after the immobilization period, thus allowing us to study the recovery phase after atrophy.

### After 1 week of recovery mice are still atrophic

**Fig 7:**
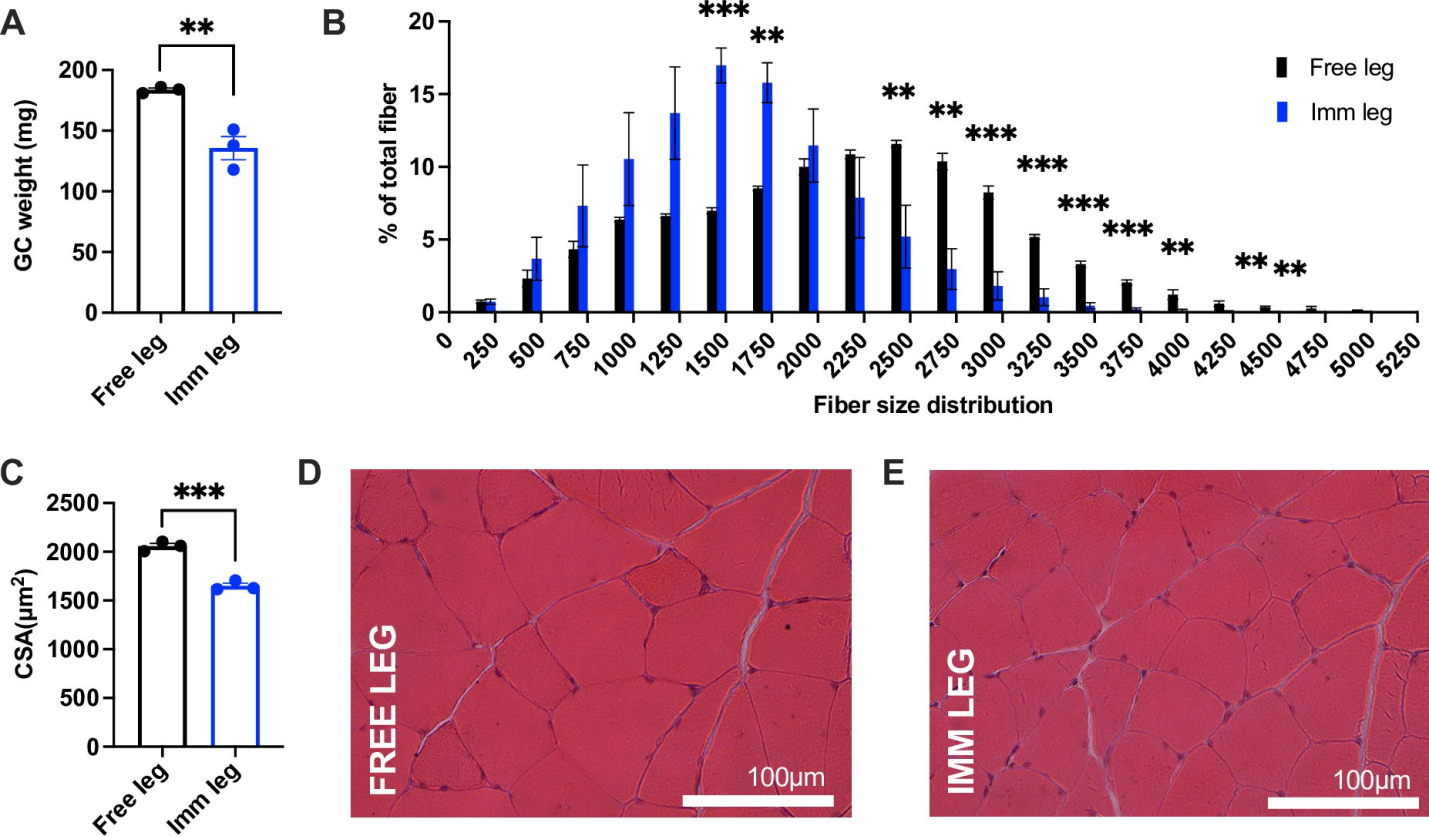
After 1 week of recovery mice are still atrophic. **(A)** Comparison between free and immobilized gastrocnemius wet weight. **(B)** Fiber size distribution (n = 3, mean±SD) and **(C)** myofibers cross-sectional area analysis of free and immobilized legs (leg n = 3 per each condition). **(D)** Representative images of Hematoxylin and Eosin of free and **(E)** immobilized gastrocnemius muscles. Scale bar = 100μm; magnification = 20x. Statistical significance was calculated using two-tailed Student’s *t* test. Data are mean±SEM (A and C), mean±SD (B). ** p<0,01 *** p<0,001.

To track the recovery phase, we harvested muscles after 1-week post-immobilization. The previously immobilized legs were still exhibited atrophy, as indicated by gastrocnemius wet weight and cross-sectional area and distribution (Fig 7A–7C), suggesting that mice require a longer period to recover fully. Histological observations revealed no alterations during the early recovery process (Fig 7D and 7E).

### After 3 weeks of reloading mice recovered completely

**Fig 8:**
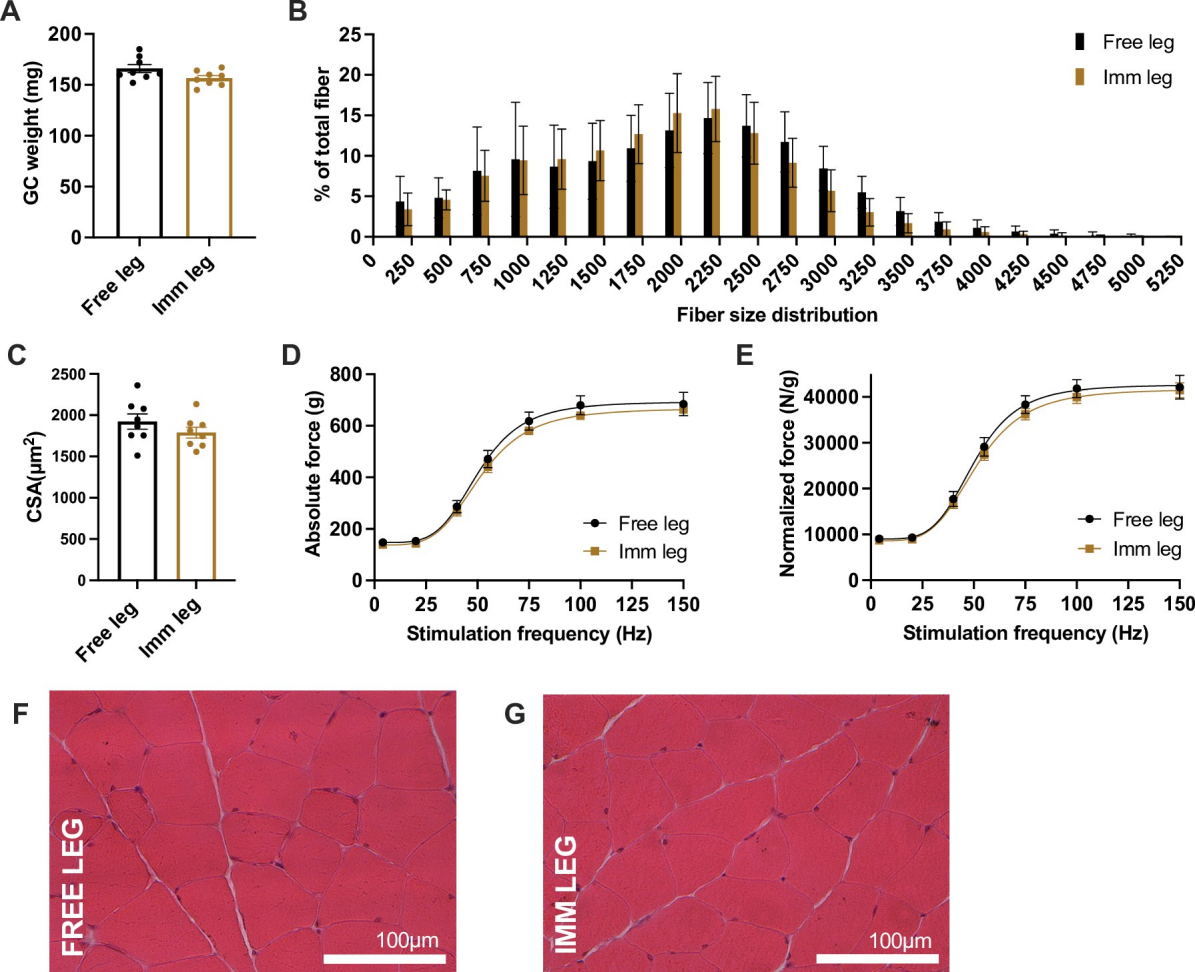
After 3 weeks of re-loading mice recovered completely. **(A)** Comparison between free and immobilized gastrocnemius wet weight. **(B)** Fiber size distribution (n = 8, mean±SD) and **(C)** myofibers cross-sectional area analysis of free and immobilized legs. **(D)** Absolute force-frequency *in vivo* on gastrocnemius muscles and **(E)** specific force generated tetanic contraction of free leg and immobilized one (leg n = 8 per each condition). **(F)** Representative images of Hematoxylin and Eosin of free and **(G)** immobilized gastrocnemius muscles. Scale bar = 100μm; magnification = 20x. Statistical significance was calculated using paired two-tailed Student’s *t* test. Data are mean±SEM (A, C, D, E), mean±SD (B).

The recovery process continued for two additional weeks, and we collected the muscle tissue three weeks after removing the cast at the end of the immobilization protocol. During this period, the legs previously subjected to immobilization fully regained their wet muscle weight (Fig 8A), cross-sectional area, and fiber size distribution (Fig 8B and 8C) and absolute and relative force (Fig 8D and 8E), indicating that our method allows for a complete recovery, avoiding long-term alterations. We confirmed this also with histological analysis of muscle slices, which showed no signs of alterations (Fig 8F and 8G). Accordingly, also the soleus muscle recovered wet muscle weight and cross-sectional area (S5 Fig).

## Discussion

The prevalence of skeletal muscle atrophy in contemporary societies underscores the need for reliable and reproducible animal models to investigate this process, as well as potential therapies and drugs. Skeletal muscle atrophy carries substantial implications for health and healthcare costs, especially in individuals unable to engage in physical exercise. Given the limited treatment options available, comprehending the underlying mechanisms and developing effective interventions becomes paramount. While existing models of disuse muscle atrophy offer advantages, they also come with limitations. In addressing the challenges of replicating human skeletal muscle disuse conditions for research, an experimental animal model known as "hindlimb suspension" (or hindlimb unloading) was introduced in the 1970s. This method, applied to rats and mice, mimics the effects of space flight and bed rest, in humans, providing an alternative avenue to study mechanisms associated with the reduction of skeletal muscle mass and to explore interventions aimed at alleviating atrophy resulting from hindlimb unloading. This protocol facilitates the examination of bone quality and the assessment of various physiological parameters, such as blood pressure, heart rate, plasma or tissue lipid composition and glycemia. Over the decades, this method has undergone several changes to reduce animal stress response, improving the reliability of the data [15, 25, 26]. However, it is important to note that this model comes with challenges, including the absence of internal controls, physical and psychological animal discomfort that may introduce variability and impede recovery studies.

Ankle joint immobilization is an alternative method to induce disuse skeletal muscle atrophy. It restricts the free movement of one of the hind limbs, which prevents the animal from normally using the immobilized leg, often resulting in a three-legged gait [22, 23, 29, 51–53]. While hind limb suspension in rats has been extensively explored in the literature, there are comparatively fewer studies focusing on unilateral models and general methods for mice. However, the use of mouse models, particularly in aging studies, is more prevalent. This underscores the need for well-established and reliable methodologies to investigate muscle atrophy in these contexts. Furthermore, several ankle joint immobilization methods require experience during the application procedure to be reproducible, and they are poorly customizable, invasive, and may induce a significant degree of inflammation.

Addressing these limitations led to the development of a novel 3D-printable plastic boot that can reliably restrict the movement of mouse hindlimbs, offering a unilateral, safe, and reproducible approach to induce muscle atrophy.

This boot allows the animals to roam their cages freely, preventing them from loading the immobilized hindlimb while avoiding the overload of the free one. Importantly, within 2 weeks, the immobilization protocol minimizes swelling and skin damage while consistently inducing muscle atrophy, as shown by decreased wet muscle weight, cross-sectional area, and muscle force. The design of the boot locks the ankle so that the foot lies at a 90° angle with the lower leg; in this way, all the hindlimb muscles are positioned in a neutral position, reaching a homogeneous degree of atrophy. Our findings unveil a significant decline in both muscle mass and cross-sectional area of the gastrocnemius muscle already after 1 week of disuse. Specifically, we observed a 14% reduction in total muscle mass and a substantial 20% decrease in the mean cross-sectional area of the gastrocnemius muscle, reaching a notable 31% reduction at the 14-day mark. The soleus muscle exhibited an even more pronounced degree of atrophy, with a notable 34% reduction. Our model appears particularly effective compared to other studies such as hindlimb suspension which induced 10–11% decrease muscle weight in gastrocnemius and 24% in soleus after 14 days [26, 54] and ankle-joint immobilization which induced 10% to 22% of atrophy [28, 29].

Histological analysis revealed no alterations in muscle architecture, inflammation, or degeneration in both free and immobilized gastrocnemius. Importantly, no differences were found in the cross-sectional area and distribution of the free leg gastrocnemius compared to age- and sex-matched wild-type mice, indicating the absence of hypertrophy or compensation in the control leg. Previous papers already reported the absence of compensation with the unilateral models [51]. In previously published methodologies, the emphasis has primarily been on mass loss, with limited correlation to strength decline or recovery. Muscle force has been measured only in a limited number of studies involving unilaterally induced muscle atrophy [21, 55], with a single study evaluating force after muscle recovery [51]. According to our functional outcome, the gastrocnemius muscle weight loss is accompanied by a 38.5% reduction in maximal absolute force when stimulated at the highest frequency of 100Hz compared to control and free legs. The specific force (the force normalized for muscle mass), was also significantly decreased by 16.5%, suggesting that force generation is also impaired in addition to a decrease in myofilaments content [56]. Importantly, no differences were found between the free leg and the legs of controls, both in terms of force kinetics and at a frequency of 100 Hz.

In line with the previous models, we found MuRF1 and Atrogin1 were quickly induced in unloaded muscles [1, 57], but for the first time, we reported MUSA1 involvement in disuse atrophy in gastrocnemius [58, 59]. It is well-established that the mRNA expression of critical genes associated with muscle atrophy decreases by 50% during hypertrophy compared to a normotrophic control, including ubiquitin ligases MuRF1 and Atrogin1 [60]. However, in our studies, there is no observed differential expression in Atrogenes in the free legs compared to the free roaming wt legs, thus ruling out overload in the free legs of boot-wearing mice.

Moreover, our model presents a slight remodeling of neuromuscular junctions in gastrocnemius, in line with already published studies in humans [61].

Disuse does not impair all fiber types to the same extent [27, 33, 54, 55, 62, 63]. Our method, in line with previous findings, induces atrophy specifically in fast type II fibers of the gastrocnemius, while slow type I fibers remain unaffected [54]. Additionally in line with Pellegrino pubblication, there is an absence of observable shifts in myosin types during the disuse period. The soleus exhibits atrophy in both type I and IIA fibers and a notable transition of myosin isoforms from type I to type IIA as previously reported in the literature [27, 33, 54, 55].

The removal of the boot allows the study of the immobilized leg recovery process, which is relevant for the development of innovative treatments for patients recovering from prolonged muscle inactivity. Our analysis showed that mice were able to start walking on four legs as soon as their boots were removed, and no difference between previously immobilized and control hindlimbs gait was observed. Therefore, the model allows us to study the reloading phase by easily removing the cast, making it highly reproducible. One week after immobilization, the mice remained atrophic, emphasizing the need for an extended recovery period. However, after 3 weeks of reloading, the animals fully recovered, as shown by muscle weight, cross-sectional area and fiber size distribution, and muscle force in gastrocnemius. Histological analysis confirmed the absence of long-term alterations. In the soleus, we observed recovery in both muscle weight and cross-sectional area.

Our model allows to selectively induce atrophy in only one limb, leaving the contralateral leg as a robust control, and it can achieve this without the need for any surgical intervention on the animal and using materials that can be produced with a common 3D printer.

This model can be used to understand further skeletal muscle atrophy thanks to its high replicability and not being highly invasive. Indeed, the physiology of disuse skeletal muscle atrophy can be observed at different time points, including the recovery phase, to investigate the role of known and candidate factors. Furthermore, drugs and therapies can be tested with this model to investigate potential treatments during immobilization and improvements during the recovery phase of disuse.

## Supporting information

S1 Data (XLSX)

S1 Fig Effects of two-weeks immobilization on tibialis anterior and soleus muscles. **(A)** Comparison of tibialis anterior muscle wet weight of the free leg, immobilized leg, and free roaming wild type leg. **(B)** Comparison of soleus muscle wet weight of the free leg and immobilized leg and **(C)** cross-sectional area. **(D,E)** Comparison between free and immobilized legs soleus muscle cross-sectional area variations according to fiber type and **(F-G)** of fiber type composition of the muscle. Statistical significance was calculated using two- tailed Student’s *t* test. Data are mean ± SEM, * p < 0,05 *** p< 0,001.
(PDF)

S2 Fig Outcome of 2 weeks of unilateral immobilization on the gastrocnemius muscle. Representative immunostaining for NCAM expression of free leg, immobilized leg and free roaming wt leg after two weeks of immobilization in gastrocnemius **(A),** tibialis anterior **(B)**, and soleus **(C)**. Scale bar = 100μm.
(PDF)

S3 Fig **(A)** Quantitative RT-PCR of Atrogenes expression in immobilized, free and free roaming wild type leg after 1 week of immobilization, normalized on actin. **(B)** Quantitative RT-PCR of NMJ genes expression in immobilized, free and free roaming wild type leg after 1 week of immobilization, normalized on actin. **(C)** Quantitative RT-PCR of Atrogenes expression in immobilized, free and free roaming wild type leg after 2 weeks of immobilization, normalized on actin. **(D)** Quantitative RT-PCR of NMJ genes expression in immobilized, free and free roaming wild type leg after 2 weeks of immobilization, normalized on actin (n = 5). Statistical significance was calculated using paired two-tailed Student’s *t* test. Data are mean±SEM, ** p<0,01 *** p<0,001.
(PDF)

S4 Fig Complete DigiGait analysis—hindlimb. Components of the stride: **(A)** %swing stride, **(B)** % brake stride, **(C)** % propel stride, **(D)** % stance stride, **(E)** % brake stance**, (F)** % propel stance, **(G)** stance/swing ratio, **(H)** Absolute paw angle, **(I)** % shared stance, **(J)** Overlap distance, **(K)** paw placement positioning (PPP), **(L)** midline distance, **(M)** paw drag (free leg n = 8; immobilized leg n = 8). Statistical significance was calculated using paired two-tailed Student’s T-test.
(PDF)

S5 Fig Soleus muscle wet weight and myofibers cross sectional area after 3 weeks of recovery. **(A)** Soleus muscle wet weight and **(B)** cross-sectional area. Statistical significance was calculated using paired two-tailed Student’s t test. Data are expressed as mean±SEM (n = 4).
(PDF)

S1 Video Boot mounting procedure. (MP4)

S2 Video Boot unmounting procedure. (MP4)

S3 Video Recording of mice freely roaming their cage with the boot preventing usage of one hindlimb, just a few minutes after boot application. (MP4)

S4 Video Recording of a mouse obtained with the DigiGait setup after boot removal. (MP4)
