# Supplementary material for: Custom-made 3D-printed boot as a model of disuse-induced atrophy in murine skeletal muscle
Source: PLoS One. 2024 May 31;19(5):e0304380. doi: 10.1371/journal.pone.0304380 (PMC11142711; doi:10.1371/journal.pone.0304380)
Supplement: S1 Fig — (A) Comparison of tibialis anterior muscle wet weight of the free leg, immobilized leg, and free roaming wild type leg. (B) Comparison of soleus muscle wet weight of the free leg and immobilized leg and (C) cross-sectional area. (D,E) Comparison between free and immobilized legs soleus muscle cross-sectional area variations according to fiber type and (F-G) of fiber type composition of the muscle. Statistical significance was calculated using two- tailed Student’s t test. Data are mean ± SEM, * p < 0,05 *** p< 0,001. (PDF) [file pone.0304380.s002.pdf]

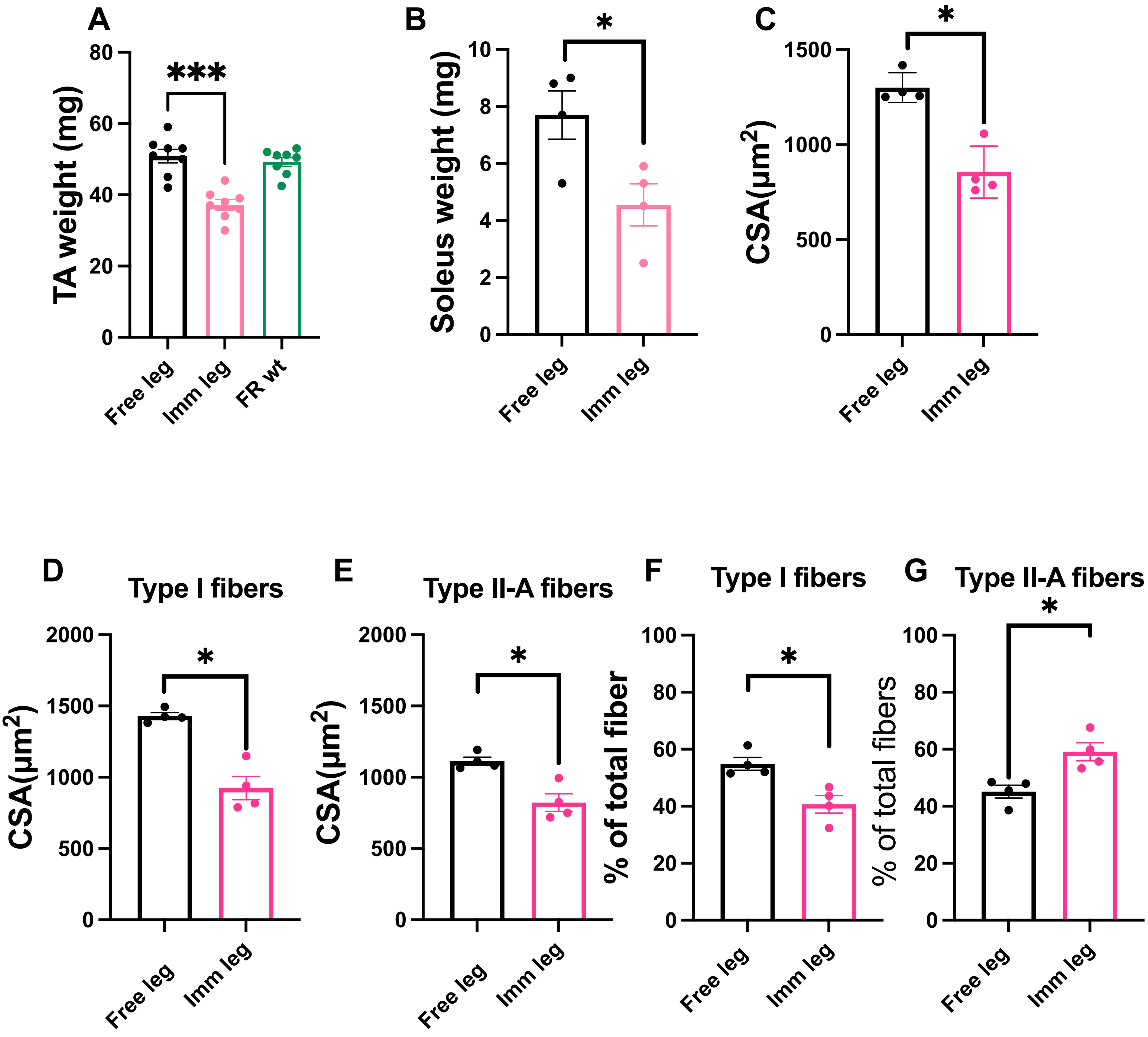

S1 Fig. Effects of two-weeks immobilization on tibialis anterior and soleus muscles. (A) Comparison of tibialis anterior muscle wet weight of the free leg, immobilized leg, and free roaming wild type leg. **(B)** Comparison of soleus muscle wet weight of the free leg and immobilized leg and **(C)** cross-sectional area. **(D,E)** Comparison between free and immobilized legs soleus muscle cross-sectional area variations according to fiber type and **(F-G)** of fiber type composition of the muscle. Statistical significance was calculated using two-tailed Student's *t* test. Data are mean ± SEM, * p < 0,05 *** p< 0,001.
