# Supplementary material for: Custom-made 3D-printed boot as a model of disuse-induced atrophy in murine skeletal muscle
Source: PLoS One. 2024 May 31;19(5):e0304380. doi: 10.1371/journal.pone.0304380 (PMC11142711; doi:10.1371/journal.pone.0304380)
Supplement: S2 Fig — Representative immunostaining for NCAM expression of free leg, immobilized leg and free roaming wt leg after two weeks of immobilization in gastrocnemius (A), tibialis anterior (B), and soleus (C). Scale bar = 100μm. (PDF) [file pone.0304380.s003.pdf]

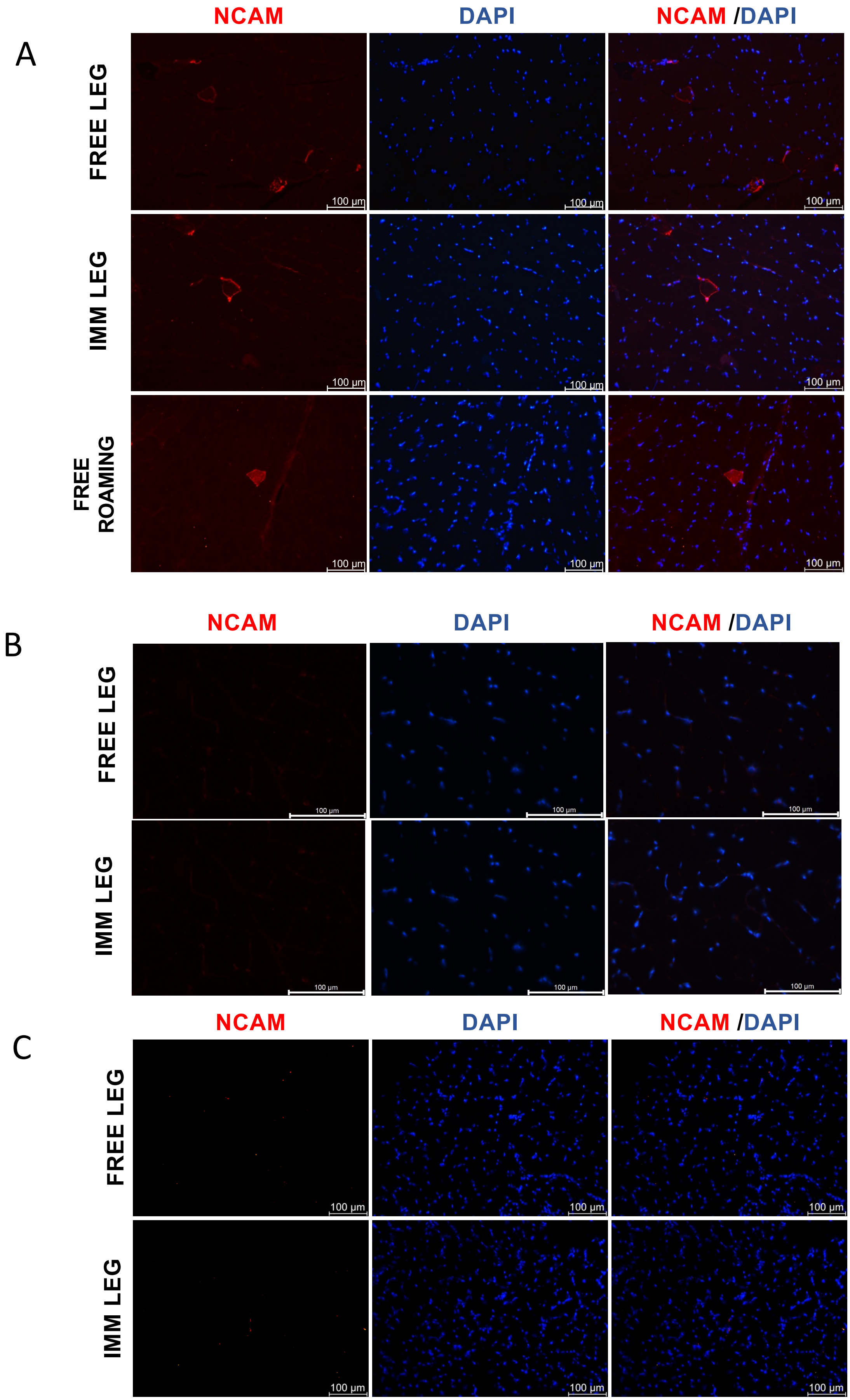

S2 Fig. Outcome of 2 weeks of unilateral immobilization on the gastrocnemius muscle. Representative immunostaining for NCAM expression of free leg, immobilized leg and free roaming wt leg after two weeks of immobilization in gastrocnemius **(A),** tibialis anterior **(B)**, and soleus **(C)**. Scale bar=100µm.
