# Supplementary material for: Custom-made 3D-printed boot as a model of disuse-induced atrophy in murine skeletal muscle
Source: PLoS One. 2024 May 31;19(5):e0304380. doi: 10.1371/journal.pone.0304380 (PMC11142711; doi:10.1371/journal.pone.0304380)
Supplement: S3 Fig — (A) Quantitative RT-PCR of Atrogenes expression in immobilized, free and free roaming wild type leg after 1 week of immobilization, normalized on actin. (B) Quantitative RT-PCR of NMJ genes expression in immobilized, free and free roaming wild type leg after 1 week of immobilization, normalized on actin. (C) Quantitative RT-PCR of Atrogenes expression in immobilized, free and free roaming wild type leg after 2 weeks of immobilization, normalized on actin. (D) Quantitative RT-PCR of NMJ genes expression in immobilized, free and free roaming wild type leg after 2 weeks of immobilization, normalized on actin (n = 5). Statistical significance was calculated using paired two-tailed Student’s t test. Data are mean±SEM, ** p<0,01 *** p<0,001. (PDF) [file pone.0304380.s004.pdf]

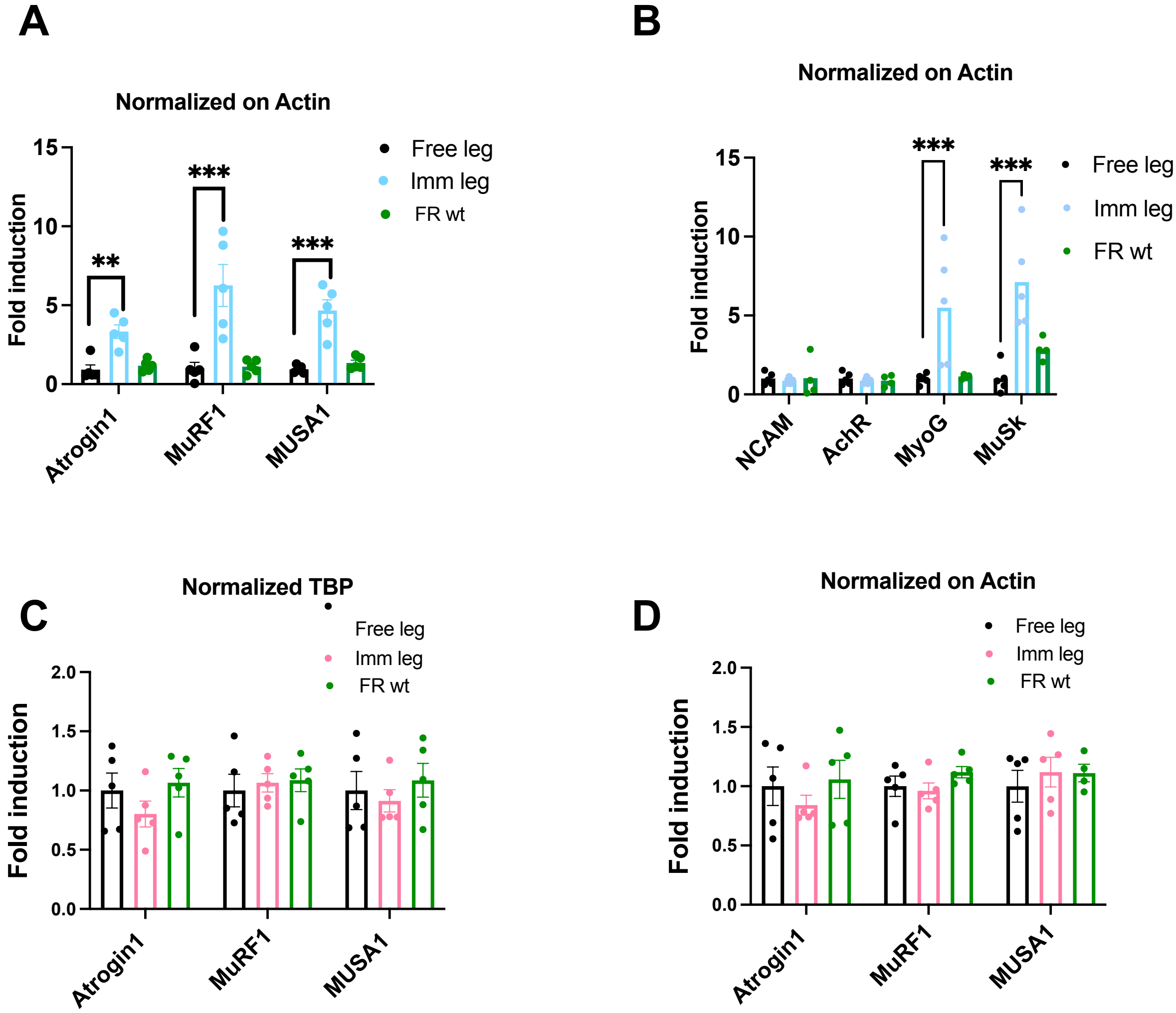

S3 Fig. (A) Quantitative RT-PCR of Atrogenes expression in immobilized, free and free roaming wild type leg after 1 week of immobilization, normalized on actin. **(B)** Quantitative RT-PCR of NMJ genes expression in immobilized, free and free roaming wild type leg after 1 week of immobilization, normalized on actin. **(C)** Quantitative RT-PCR of Atrogenes expression in immobilized, free and free roaming wild type leg after 2 weeks of immobilization, normalized on actin. **(D)** Quantitative RT-PCR of NMJ genes expression in immobilized, free and free roaming wild type leg after 2 weeks of immobilization, normalized on actin (n=5). Statistical significance was calculated using paired two-tailed Student's *t* test.

Data are mean±SEM, ** p<0,01 *** p<0,001.
