# Supplementary material for: Custom-made 3D-printed boot as a model of disuse-induced atrophy in murine skeletal muscle
Source: PLoS One. 2024 May 31;19(5):e0304380. doi: 10.1371/journal.pone.0304380 (PMC11142711; doi:10.1371/journal.pone.0304380)
Supplement: S4 Fig — Components of the stride: (A) %swing stride, (B) % brake stride, (C) % propel stride, (D) % stance stride, (E) % brake stance, (F) % propel stance, (G) stance/swing ratio, (H) Absolute paw angle, (I) % shared stance, (J) Overlap distance, (K) paw placement positioning (PPP), (L) midline distance, (M) paw drag (free leg n = 8; immobilized leg n = 8). Statistical significance was calculated using paired two-tailed Student’s T-test. (PDF) [file pone.0304380.s005.pdf]

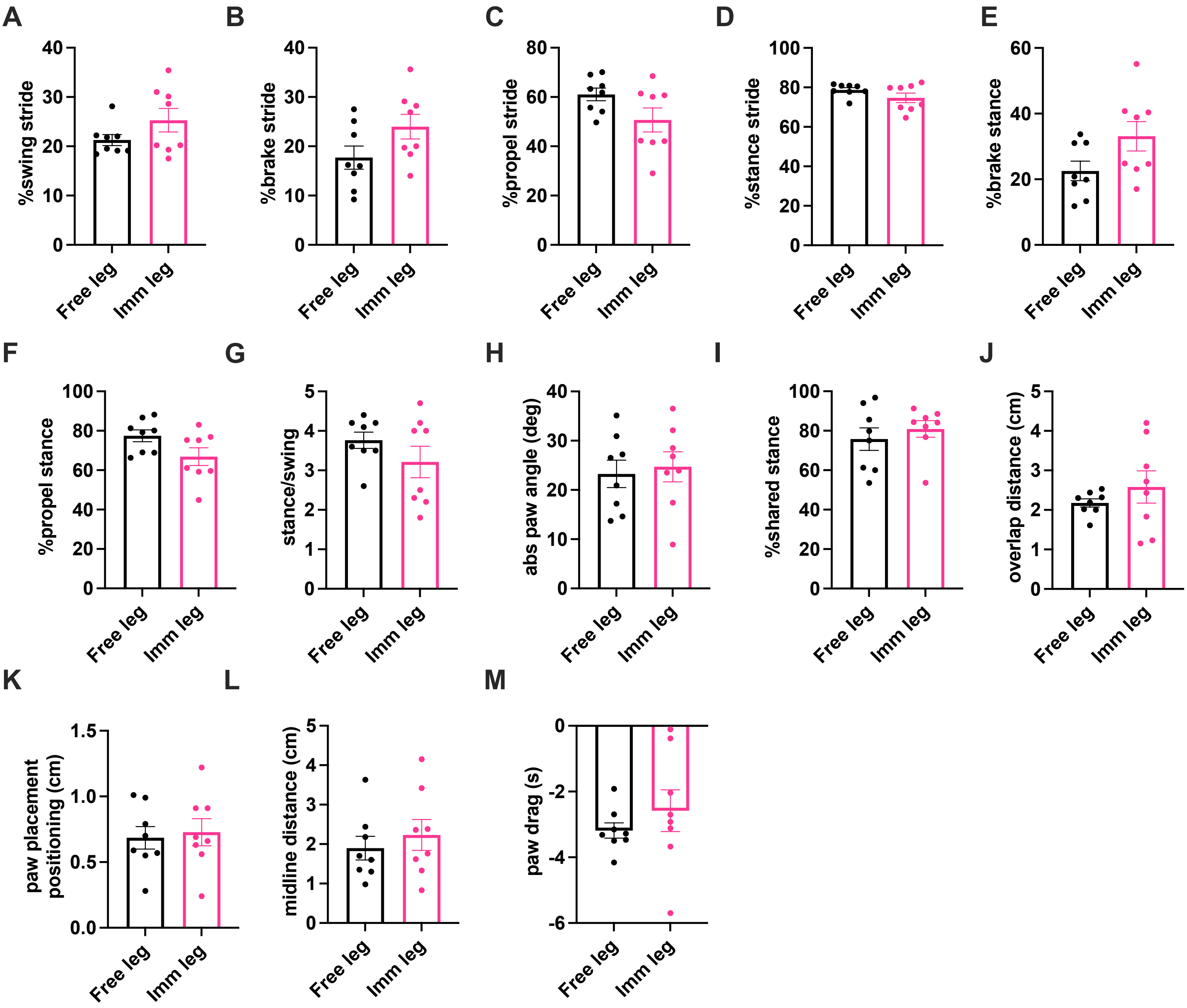

Supp Fig 4. Complete DigiGait analysis - hindlimb. Components of the stride: **(A)** %swing stride, **(B)** % brake stride, **(C)** % propel stride, **(D)** % stance stride, **(E)** % brake stance**, (F)** % propel stance, **(G)** stance/swing ratio, **(H)** Absolute paw angle, **(I)** % shared stance, **(J)** Overlap distance, **(K)** paw placement positioning (PPP), **(L)** midline distance, **(M)** paw drag (free leg n=8; immobilized leg n=8). Statistical significance was calculated using paired two-tailed Student's T-test.
