# Supplementary material for: Custom-made 3D-printed boot as a model of disuse-induced atrophy in murine skeletal muscle
Source: PLoS One. 2024 May 31;19(5):e0304380. doi: 10.1371/journal.pone.0304380 (PMC11142711; doi:10.1371/journal.pone.0304380)
Supplement: S5 Fig — (A) Soleus muscle wet weight and (B) cross-sectional area. Statistical significance was calculated using paired two-tailed Student’s t test. Data are expressed as mean±SEM (n = 4). (PDF) [file pone.0304380.s006.pdf]

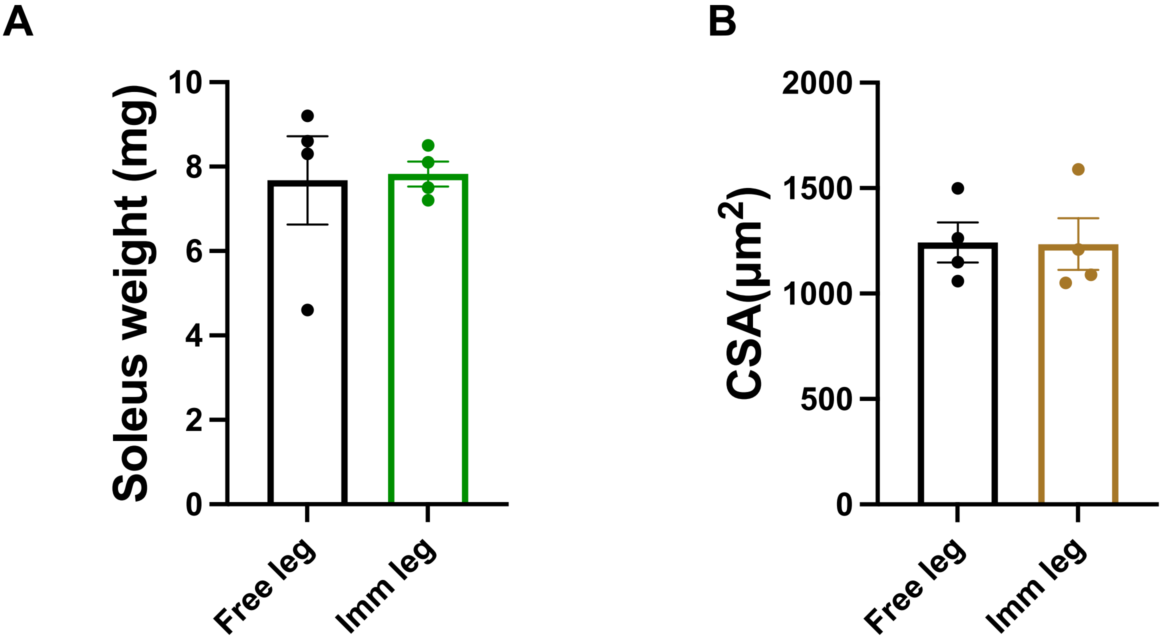

S5 Fig. Soleus muscle wet weight and myofibers cross sectional area after 3 weeks of recovery. (A) Soleus muscle wet weight and **(B)** cross-sectional area. Statistical significance was calculated using paired two-tailed Student's t test. Data are expressed as mean±SEM (n=4).
